# Twenty Years of Research on Mineral Trioxide Aggregate: A Scientometric Report

**Published:** 2013-01-20

**Authors:** Saeed Asgary, Hamid Reza Motazedian, Masoud Parirokh, Mohammad Jafar Eghbal, Sanam Kheirieh

**Affiliations:** 1 Iranian Center for Endodontic Research, Research Institute of Dental Sciences, Shahid Beheshti University of Medical Sciences, Tehran, Iran; 2 Oral and Dental Diseases Research Center, Kerman University of Medical Sciences, Kerman, Iran; 3 Dental Research Center, Research Institute of Dental Sciences, Shahid Beheshti University of Medical Sciences, Tehran, Iran

**Keywords:** Biomaterial, Endodontic, Mineral trioxide aggregate, MTA, PubMed, Scientometric

## Abstract

**Introduction:**

Mineral trioxide aggregate (MTA) has been suggested for root-end filling, vital pulp therapy, apical plug, perforations repair, and root canal filling. Since the introduction of MTA in 1993, many studies about this material have been published. The aim of this survey was to illustrate statistical information about published articles in PubMed-index journals vis-à-vis the various aspects of this biomaterial.

**Material and Methods:**

A PubMed search was performed to retrieve the relative articles from 1993 to August 2012. The data of each article including publication year, journal name, number of authors, first author name, affiliations and study design were recorded. Citation of each article till 2009 was obtained from Scopus and Google scholar databases. Data were analyzed to determine the related scientometric indicators.

**Results:**

In total, 1027 articles were found in PubMed-indexed journals which show considerable increase from 2 papers in 1993 to 139 in 2011. While ~62% of articles had no level of evidence, only ~5% could be classified as having the highest level of evidence (LOE1); however, the majority of LOE1 articles originated from Iran (~1%: n=10). Journal of Endodontics, as the top rank journal, published 31.7% of MTA related articles. The majority of articles were four-authored (19.6%). Most of the articles originated from USA (21.9%), Brazil (18.5%) and Iran (8.76%). The average number of citation for the top ten articles from Scopus was 231.

**Conclusion:**

This data demonstrates that during the past two decades, research on this novel endodontic biomaterial had a rapid positive trend especially during the last 5 years. Further high-level evidence articles for the various clinical applications of MTA would result in superior clinical decision making and stronger scientific-based endodontic practice.

## 1. Introduction

Research investigations in endodontic science are mostly focused on preservation of pulp tissue and regenerative endodontics, they are also concerned with the eradication of bacteria from the root canal and sealing the pathways between root canal spaces and periradicular tissues. There is no doubt that the introduction of mineral trioxide aggregate (MTA) by Torabinejad in 1993 has made a great impact in endodontic practice and has improve the success rate in every dental office all around the world. MTA was first introduced as a water-based gray-colored root-end filling and perforation repair material [1]. Because of the potential discoloration effect of grey MTA, a tooth-colored formula has been introduced in new millennium [2]. Lime (CaO), silica (SiO2), and bismuth oxide (Bi2O3) are the major oxides in both types of MTA [3]. It has high pH but low compressive strength; depending on its powder/liquid ratio, MTA possesses some antibacterial/fungal properties [4]. MTA seals well and is biocompatible cement; hydroxyapatite crystals form over MTA in contact with tissue fluid [5, 6]. However, it has some known drawbacks such as a long setting time, high price, and potential discoloration [7].

The amount of new medical/dental information is drastically increasing; doubling time for medical knowledge, by 1991 standards, had been estimated to be 19 years [8]; however, in 2010 this period was estimated for only 3.5 years. Interestingly, in 2020 it is projected to be ~2.5 months [9]. While during a decade after introduction of MTA more than 120 articles were published on the properties and applications of this novel material [3], in 2010 a three part comprehensive literature review by Parirokh and Torabinejad evaluated ~700 published articles on MTA [4, 5, 7]. Dental clinicians need information to keep up-to-date with new progresses applicable to clinical practice as well as to seek for answers to patients’ problems. The exponential growth in biomedical knowledge has shifted dental clinicians towards a new paradigm, i.e. evidence based dentistry by using highest level of evidences (LOE) [10].

We aimed to evaluate the quantity as well as quality of research articles in PubMed-indexed journals with respect to mineral trioxide aggregate from the introduction of the material in 1993 until now.

## 2. Material and Methods

In order to collect the articles, mineral trioxide aggregate was used as the keyword. Number of published articles in PubMed-indexed journals was determined without time limitation at the search time (August 2012). Abstracts were reviewed and only related articles were included. Papers were classified in respect to the study design. The data of each article including the publication year, journal name, number of authors, first author name, and affiliations were recorded. Number of citations was also determined from Scopus and Google Scholar. For top ten authors, the H-Index of them from Scopus was also traced.

## 3. Results

**Figure 1:**
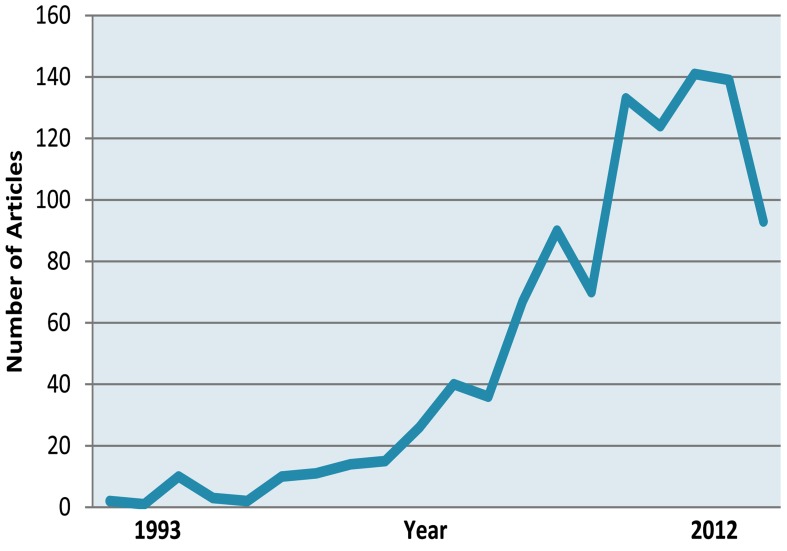
Trend of MTA’s published articles in PubMed-indexed journals from 1993 until July 2012

Overall, 1027 articles were published in the PubMed-indexed journals regarding MTA (Table 1); Figure 1 shows positive trends in number of published articles during past 20 years. Torabinejad, as the inventor of MTA, owned 17 articles as first author; he also contributed in 22 articles as co-author.

**Table 1:** Distribution of published articles from 1993 to July 2012

	Year	Number (%)	Torabinejad	
			First Author	Co-author
1	****1993****	**2 (0.19)**	**1**	**1**
2	**1994**	**1 (0.09)**	**1**	**0**
3	**1995**	**10 (0.97)**	**7**	**2**
4	**1996**	**3 (0.29)**	**0**	**2**
5	**1997**	**2 (0.19)**	**1**	**1**
6	**1998**	**10 (0.97)**	**1**	**2**
7	**1999**	**11 (1.07**)	**1**	**2**
8	**2000**	**14 (1.36)**	**0**	**1**
9	**2001**	**15 (1.46)**	**0**	**1**
10	**2002**	**26 (2.53)**	**0**	**2**
11	**2003**	**40 (3.89)**	**0**	**1**
12	**2004**	**36 (3.50)**	**1**	**3**
13	**2005**	**67 (6.52)**	**0**	**0**
14	**2006**	**90 (8.76)**	**0**	**0**
15	**2007**	**70 (6.81**)	**0**	**0**
16	**2008**	**133 (12.95)**	**0**	**0**
17	**2009**	**124 (12.07)**	**2**	**2**
18	**2010**	**141 (13.72)**	**1**	**2**
19	**2011**	**139 (13.53)**	**1**	**0**
20	**2012**	**93 (9.05)**	**0**	**0**
	**Total**	**1027**	**17**	**22**

Table 2 shows the top 10 journals with the highest number of publications regarding MTA. While the Journal of Endodontics stands as top-ranked, International Endodontic Journal had published the highest number of LOE1 articles. The Impact Factor of the journals in 2010 is also presented.

**Table 2:** Top Ten Journals

	Journal name (Impact Factor of 2010)	Number of articles	Number of LOE 1
1	JEndod(3.291)	**326**	**4**
2	IntEndodJ (2.383)	**128**	**10**
3	OralSurgOral Med OralPatholOralRadiolEndod(1.417)	**91**	**3**
4	DentTraumatol(1.268)	**42**	**0**
5	AustEndodJ (1.239)	**25**	**0**
6	BrazDent J (-)	**17**	**0**
7	PediatrDent (1.831)	**17**	**5**
8	JApplOralSci(0.966)	**16**	**0**
9	Dent Mater (1.112)	**16**	**0**
10	J OralSci(-)	**15**	**0**

The number of authors varied from 1 to 12; however, the majority of articles (19.6%) were written by four authors. The average number of authors for all of these scientific publications was ~4 (Table 3).

**Table 3:** Number of authors

Number of authors	#Article
1	**101**
2	**135**
3	**187**
4	**202**
5	**155**
6	**132**
7	**58**
8	**33**
9	**12**
10	**6**
11	**1**
12	**1**

Table 4 shows the top 12 first authors with the highest number of publications. Top three first authors from Malta, USA and Iran had produced >10 publications; their H-index are also presented in the Table.

**Table 4:** Top 12 first authors

	Name of authors	Country	# Article	H-index
1	CamilleriJ	Malta	**23**	**12**
2	TorabinejadM	USA	**17**	**28**
3	AsgaryS	Iran	**12**	**15**
4	SaghiriMA	Iran	**9**	**5**
5	Holland R	UK	**8**	**15**
6	GandolfiMG	Italy	**8**	**14**
7	Gomes-FilhoJE	Brazil	**8**	**7**
8	YildirimT	Turkey	**7**	**7**
9	Al-HezaimiK	Saudi Arabia	**7**	**6**
10	ParirokhM	Iran	**6**	**14**
11	NekoofarMH	Iran	**6**	**7**
12	ShahiS	Iran	**6**	**5**

**Figure 2:**
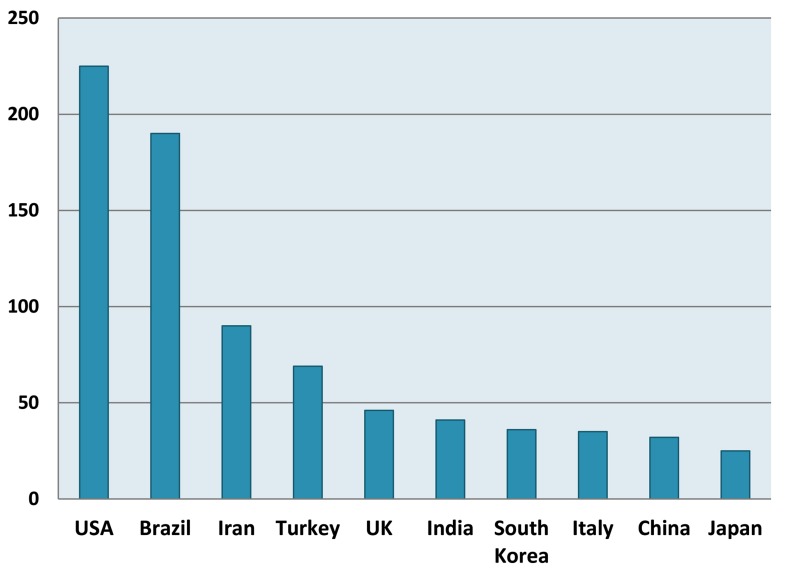
Top ten Countries

In respect to nationality categorizing (Figure 2), majority of publications belonged to the United States of America (21.9%) followed by Brazil (18.5%), and Iran (8.76%).

**Figure 3:**
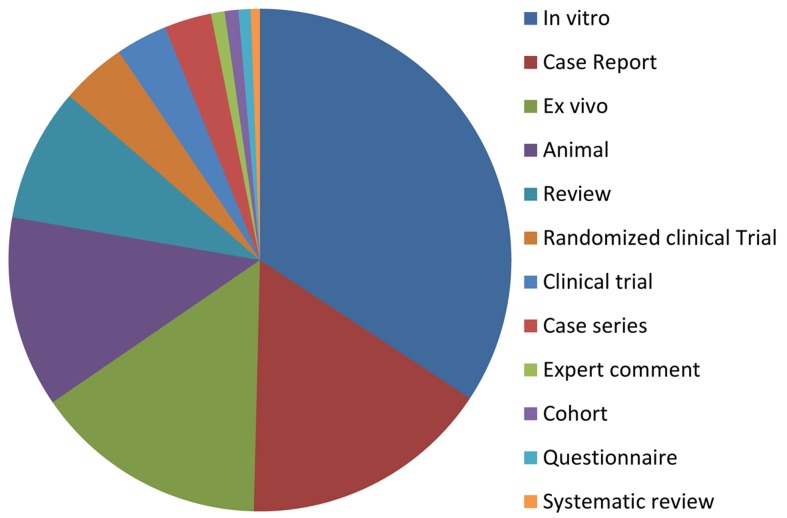
Distributions of MTA’s articles regarding their study design

**Figure 4:**
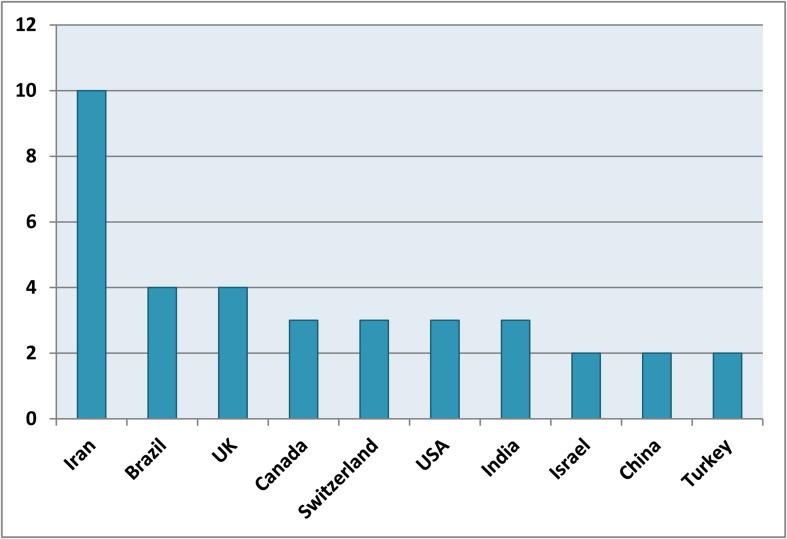
Distributions of LOE1 articles

The greatest portion of articles (49.1%) was related to in vitro/ex vivo studies; while, the least belonged to systematic reviews (0.5%). Figure 3 shows the distribution of articles regarding their study design. Majority of LOE1 articles originated from Iran, Brazil and UK (Figure 4). The average of citation from Scopus for the top 10 articles was 231 (Table 5).

**Table 5:** Scopus/Google citations for the top 10 Articles

Author/Journal/Year/Title	Google	Scopus
Torabinejad(JEndod; 1993): *Sealing ability of a mineral trioxide aggregate when used as a root end filling material.*	**680**	**369**
Lee (J Endod;1993): *Sealing ability of a mineral trioxide aggregate for repair of lateral root perforations.*	**651**	**336**
Torabinejad(J Endod;1994): *Dye leakage of four root end filling materials: effects of blood contamination.*	**581**	**308**
Torabinejad(J Endod;1995): *Investigation of mineral trioxide aggregate for root-end filling in dogs.*	**491**	**247**
Torabinejad(J Endod;1995): *Tissue reaction to implanted super-EBA and mineraltrioxide aggregate in the mandible of guinea pigs: a preliminary report.*	**351**	**201**
Ford (Triple O;1995): *Use of mineral trioxide aggregate for repair offurcalperforations.*	**329**	**183**
Kettering (J Endod;1995): *Investigation of mutagenicity of mineral trioxide aggregate and other commonly used root-end filling materials.*	**326**	**186**
Torabinejad(J Endod;1995): *Cytotoxicity of four root end filling materials.*	**312**	**169**
Torabinejad(J Endod;1995): *Antibacterial effects of some root end filling materials.*	**311**	**156**
Torabinejad(J Endod;1995): *Physical and chemical properties of a new root-end filling material.*	**296**	**155**

## 4. Discussion

In this study, the articles were retrieved using the PubMed database; an inclusive database run by the National Library of Medicine. The database was selected to search through the biomedical literature because it is found to be comprehensive/representative in its coverage, freely available and extremely up-to-date; at present PubMed comprises more than 22 million citations for biomedical literature from MEDLINE, life science journals, and online books [11].

This study was performed to provide statistical information about published articles in PubMed-index journals regarding MTA quantitatively and qualitatively. A noticeable enhance of interest in MTA research is evident worldwide as reflected by the continuously rising number of publications since the introducing of the biomaterial in 1993; however, until the FDA approval for the material in (1998), majority of researches were conducted by MTA’s inventor.

Currently, IF as well as abstracting/indexing in the leading catalogues and indexing systems are basic factors for ranking of dental journals. The Journal of Endodontics is ranked 3rd out of 74 journals in the Dentistry, Oral Surgery and Medicine category on the 2010 Journal Citation Reports, published by Thomson Reuters, and has an Impact Factor of 3.291; it is indexed in PubMed and ISI as well. The Journal of Endodontics as the first rank endodontic journal has published 31.74% of articles discussing MTA.

According to the findings of this survey, the total number of authors that wrote about MTA was 4168. The average number of authors per paper is ~4. The number of single-authored articles accounts for ~10%; however, this percent for four-authored articles is 19.6%. These data indicating growing tendency towards collaboration and increasing the group size in team workings.

In this study, in order to evaluate those first authors of articles related to MTA who has the most involvement in this subject, the number of published items (index of productivity) and the h-index (index of quality) belonged to them were determined and reported. According to this report, top three first authors were from Malta, USA and Iran. Moreover, data analysis showed that the researchers from USA, Brazil and Iran conducted the majority of MTA researches and their countries keep a leading position in MTA research.

In vitro, ex vivo and animal studies are moderately easy to do, low-priced to complete; they also provide a significant amount of data. These studies are relatively fast when compared to other bioassay procedures, and can be carried out in controlled conditions. On the other hand, randomized controlled clinical trials (RCTs) are difficult, expensive, need long term follow-up, and require skilled methodologist. RCTs have long been considered the “gold standard” for assessing the efficacy/safety of medical/dental agents; results of well-designed RCTs can be generalized due to principles of evidence based practice [10]. Our results demonstrated that there are 49 RCTs and systematic reviews regarding MTA (<5%); these studies are considered as first level of evidence (LOE). However, the majority of published studies were in vitro, ex vivo and animal studies (~62%). Obviously, according to principles of evidence based dentistry /endodontics the results of these types of study do not have clinical relevancy. Parirokh and Torabinejad in their comprehensive review (Part III) also expressed their concern that despite employing MTA for several years in dentistry and endodontics, few clinical investigations with high level of evidence were performed compared to the laboratory and animal studies. Thus, they recommended that future investigations should focus on clinical applications of the material and its success rate in pulp capping, root-end filling, apical plug, and perforation repair [7]. Bakland and Andreasen, through their recent review on replacing calcium hydroxide with MTA as a material of choice for pulp capping, stated that due to that lack of long-term clinical trials that give enough documents for supporting that idea more investigations with high level of evidence are recommended in that regard [12].

The quantity of new (bio)dental data/information is incredible; not all published ones are valid or even relevant to dental clinicians. Dental research needs to be filtered and appraised/summarized before it can be applied in practice. Published studies with well designed methodology as well as highest ranked LOE can have significant impact on evidence based clinical recommendation of MTA as a novel generation of endodontic biomaterials worldwide. Although MTA has been introduced, approved and commercialized in USA by Torabinejad (an Iranian scientist), at the present time, only 3 LOE1 articles originated from USA. Although the number of endodontic articles in PubMed indexed journal from Iran is rapidly increased in recent years [13] and this country is in the second rank for endodontic science productivity in Middle East region [14], Iran does not produce the greatest number of LOE endodontic articles [15]; but interestingly Iran is the first ranked country which conducted >10% of all LOE1 articles for MTA.

Numerous in vitro and in vivo articles have been published regarding various aspects of MTA. Correlation between laboratory results and clinical performance of a biomaterial has frequently been questioned [16]. Most of those studies show a material with promising results; however, it has been generally accepted that the lack of evidence-based literature is a major drawback for MTA [17]. Therefore, we strongly recommend further clinical studies for various applications of MTA to support its ability as a root-end filling, root-end barrier, as well as perforation repair and pulp capping material.

## 5. Conclusion

The current study is the first detailed scientometric analysis of MTA in dentistry. The data illustrates a noticeable increase in research productivity since the 1993. While the majority of articles originate from the USA, Brazil and Iran respectively, Maltese, American and Iranian scientists were the top ranked first authors with high impact articles. Iran conducted the majority of LOE1 articles about MTA and is the leading country for conducting randomized clinical trials.
