# Twenty Years of Research on Mineral Trioxide Aggregate: A Scientometric Report

**Published:** 2013-05-01

**Authors:** Saeed Asgary, Hamid Reza Motazedian, Masoud Parirokh, Mohammad Jafar Eghbal, Sanam Kheirieha

**Affiliations:** 1 Iranian Center for Endodontic Research, Research Institute of Dental Sciences, Shahid Beheshti University of Medical Sciences, Tehran, Iran; 2 Oral and Dental Diseases Research Center, Kerman University of Medical Sciences, Kerman, Iran; 3 Dental Research Center, Research Institute of Dental Sciences, Shahid Beheshti University of Medical Sciences, Tehran, Iran

In the article “Table 4. Top 12 first authors” was published with errors; the corrected Table is as follows:

**Table 4:** Top 12 first authors

	Name of authors	Country	# article	H-index
1	Camilleri J	Malta	**23**	**12**
2	Torabinejad M	USA	**15**	**28**
3	Asgary S	Iran	**13**	**15**
4	Gandolfi MG	Italy	**12**	**14**
5	Holland R	Brazil	**9**	**15**
6	Gomes-Filho JE	Brazil	**9**	**7**
7	Saghiri MA	Iran	**8**	**5**
8	Al-Hezaimi K	Saudi Arabia	**7**	**6**
9	Parirokh M	Iran	**6**	**14**
10	Yildirim T	Turkey	**6**	**7**
11	Nekoofar MH	Iran	**6**	**7**
12	Shahi S	Iran	**6**	**5**

